# Structural Basis for Specificity of Propeptide-Enzyme Interaction in Barley C1A Cysteine Peptidases

**DOI:** 10.1371/journal.pone.0037234

**Published:** 2012-05-17

**Authors:** Inés Cambra, David Hernández, Isabel Diaz, Manuel Martinez

**Affiliations:** Centro de Biotecnología y Genómica de Plantas, Universidad Politécnica de Madrid, Pozuelo de Alarcón, Madrid, Spain; Ghent University, Belgium

## Abstract

C1A cysteine peptidases are synthesized as inactive proenzymes. Activation takes place by proteolysis cleaving off the inhibitory propeptide. The inhibitory capacity of propeptides from barley cathepsin L and B-like peptidases towards commercial and barley cathepsins has been characterized. Differences in selectivity have been found for propeptides from L-cathepsins against their cognate and non cognate enzymes. Besides, the propeptide from barley cathepsin B was not able to inhibit bovine cathepsin B. Modelling of their three-dimensional structures suggests that most propeptide inhibitory properties can be explained from the interaction between the propeptide and the mature cathepsin structures. Their potential use as biotechnological tools is discussed.

## Introduction

Plant proteolysis is a complex process that involves broad metabolic networks, different sub-cellular compartments and various types of peptidases, mainly cysteine-, serine-, aspartic- and metallo-peptidases [1]. Among the about 800 peptidases encoded by plant genomes, approximately 140 correspond to cysteine-peptidases that belong to 15 families distributed in 5 clans [2]. In particular, the papain-peptidases C1A (family C1, clan CA), grouped as cathepsin L-, B-, H- and F-like according to their gene structures and phylogenetic relationship [3], are the most abundant. Members of the papain-like subfamily C1A are the most widely studied among plant cysteine peptidases. All C1A proteins contain several disulphide bonds and share three conserved catalytic residues, Cys, His and Asn, in the catalytic triad and a Gln residue involved in maintaining an active enzyme conformation. C1A peptidases from plants are synthesized as inactive or little active precursors to prevent inappropriate proteolysis. Immature proteins comprise an N-terminal propeptide of 130–160 amino acids and the mature protein domain that is about 220–270 residues long. Three main functions have been assigned to C1A propeptides: inhibition of their cognate enzyme, participation in correct intracellular targeting of the protease, and assistance in folding of the mature enzyme [4]. In this way, the pro-sequences play important roles as modulators of the peptidase activity to guarantee that the mature enzyme is formed in the right place and/or at the right time [5].

From crystal structures of procathepsins B and L from mammals [6], [7], it has been determined that the propeptide forms a predominantly α-helical domain, which is positioned at the top of the cysteine peptidase catalytic site, where it interacts with the mature part. The propeptide chain then continues in an extended conformation across the active-site cleft and towards the N-terminus of the mature enzyme in the reverse orientation to that of substrate binding. The C1A propeptides contain the consensus motif GxNxFxD, which seems to be essential for the correct processing of the peptidase precursors as well as the non-contiguous ERFNIN signature (Ex3Rx3Fx3Nx3I/Vx3N) found in cathepsin L- and H-like or the ERFNAQ variant in cathepsin F-like, both of unknown function [3], [8]. In contrast, cathepsin B-like peptidases lack this motif [3], [4].

To become active, the C1A peptidases need to be processed either autocatalytically or with the aid of processing enzymes [9]. Activation takes place by limited intra and intermolecular proteolysis cleaving off the inhibitory propeptide [10]. For most C1A cysteine peptidases, activation mechanism has been proposed to be a two steps process [11], [12]. One step corresponds to the enhancement of the accessibility to the scissile bond triggered by low pH through intramolecular conformational changes of the propeptide. The second step corresponds to the intermolecular proteolysis of the scissile bond achieved in an autocatalytic manner of by other proteases.

Selectivity of propeptide inhibition is a crucial feature to be addressed for using propeptides as biotechnological tools. In this way, many mammalian C1A propeptides not only are able to inhibit their cognate enzymes, but have the capacity to inhibit in trans several but not all related peptidases [4]. In plants, only the inhibitory capacity of propeptides from papain and papaya proteinase IV against papaya cathepsin L-like cysteine peptidases has been determined [13], [14]. In this paper, we characterize the inhibitory capacity of propeptides from barley cathepsin L and B-like peptidases towards commercial and barley cathepsins. Modelling of the three-dimensional structures suggests that most inhibitory properties can be explained from the interaction between the propeptide and mature cathepsin structures.

## Results

### Purification of propeptides and inhibitory assays

**Figure 1:**
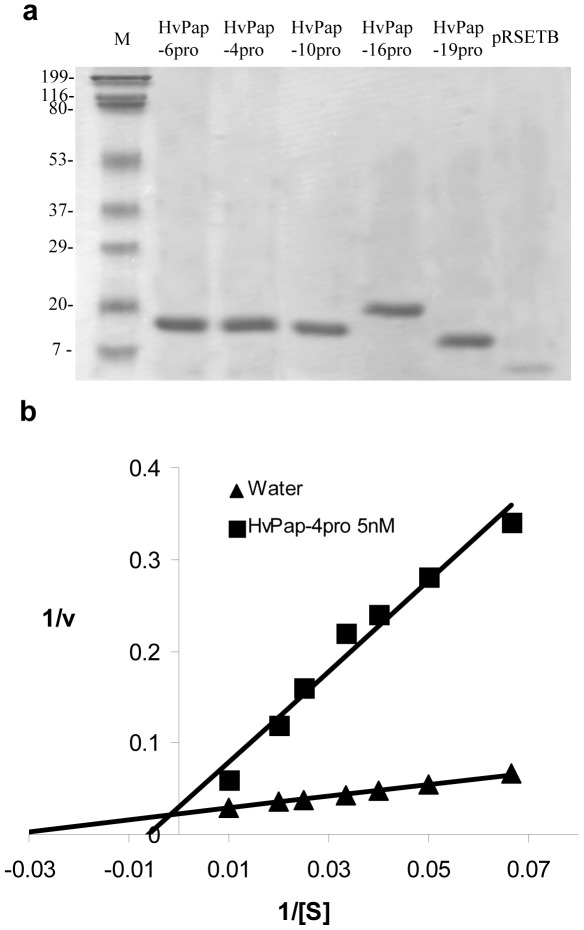
Purification and inhibitory mechanism of propeptides. A. Purification of the recombinant HvPap propeptides from *E. coli* cultures by SDS-PAGE. Five µg of each protein were loaded. Five µg of an extract of pRSETB without insert were used as a control of protein purity. The gel was stained with Coomassie Brillant Blue G. M: molecular markers (KD). **B**. Example of Lineweaver-Burk plot. HvPap-10 competitive inhibition in the presence of HvPap-4pro. Water was used as a control.

To fully characterize the inhibitory properties of C1A propeptides in barley, we selected four cathepsin L-like (HvPap-4, -6, -10, -16) peptidases and one cathepsin B-like (HvPap-19) that had been previously described [15], [16]. To study their inhibitory capability, their propeptides were purified as recombinant proteins from *E. coli* cultures. Bands detected after SDS-PAGE were in accordance with the expected molecular weights of purified propeptides, which rank from 12 kD of HvPap-19pro to 19 kD of HvPap-16pro (Fig. 1a). *In vitro* inhibitory assays against purified barley cathepsin L-like peptidases or commercial bovine cathepsin B were done using substrates able to be degraded by cathepsin L and B-like enzymes. The inhibitory activity of propeptides was not assayed against the barley cathepsin B-like HvPap-19 due to the difficulties of obtaining active purified forms from recombinant *E. coli* cultures. Kinetic analyses revealed that barley propeptides exhibited a competitive tight binding inhibition against all peptidases tested (Fig. 1b). The inhibition constant values (*K*_i_) against the C1A cysteine peptidases were determined and showed different target specificities (Table 1). Overall, the lowest *K*_i_ data that reflects the highest inhibitory capacity of the propeptides were obtained for HvPap-4pro and HvPap-6pro, which were able to inhibit all barley L-like cathepsins tested. Interestingly, both propeptides inhibit better the peptidase activity of HvPap-10 than the activities of their cognate peptidases. Likewise, HvPap-10pro and HvPap-16pro inhibit worse their maternal peptidases than HvPap-4pro and HvPap-6pro, being the inhibitory capacity of HvPap-16pro specific of its cognate peptidase. On the other hand, the propeptide of the cathepsin B-like peptidase (HvPap-19pro) was able to inhibit the activity of the cathepsin L-like peptidases HvPap-4 and HvPap-16. However, it did not inhibit the activity of the bovine cathepsin B, which was also not inhibited by any of the cathepsin L-like propeptides tested.

**Table 1:** Inhibition constants (*K*_i_) values of the barley propeptides (HvPap-4pro, -6pro, -10pro, -16pro, -19pro) against barley cathepsin L-like (HvPap-4, -6, -10, -16) and commercial cathepsin B (BovCathB) C1A cysteine peptidases.

Propeptide	HvPap-4	HvPap-6	HvPap-10	HvPap-16	BovCathB
HvPap-4pro	1.3×10^−9^	2.3×10^−9^	3.1×10^−10^	8.5×10^−9^	ni
HvPap-6pro	4.8×10^−9^	1.5×10^−9^	4.2×10^−10^	5.4×10^−9^	ni
HvPap-10pro	9.7×10^−9^	3.6×10^−8^	7.5×10^−9^	ni	ni
HvPap-16pro	ni	ni	ni	2.2×10^−8^	ni
HvPap-19pro	1.5×10^−7^	ni	ni	5.4×10^−8^	ni

ni, no inhibitory activity observed at 5 µM concentration of the propeptide.

### Structural explanation for bovine cathepsin B inhibition

**Figure 2:**
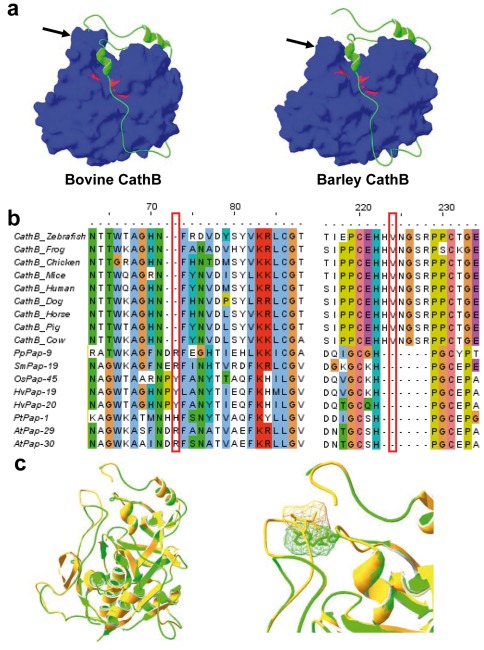
Structure-sequence analysis of animal and plant B cathepsins. (a) Homology models of bovine and barley HvPap-19 cathepsin B peptidases created using SWISS-MODEL. The propeptides (green) and the mature enzymes (blue; catalytic triad residues in red) are shown. Arrows mark the occluding loop domains. (b) Alignment of the amino acid regions involved in the interaction between the occluding loop and the propeptide in different animal and plant species. Alignment was generated using the MUSCLE program. The locations of residues potentially involved in steric clashes (red boxes) are indicated. Pp, *Physcomitrella patens*; Sm, *Selaginella moellendorffii*; Os, *Oryza sativa*; Hv, *Hordeum vulgare*; Pt, *Populus trichocarpa*; At, *Arabidopsis thaliana*. (c) Ribbon plots showing the structural overlay of three-dimensional models for bovine cathepsin B (orange) and HvPap-19 (green). Close-up image shows the molecular surface of V174 located in the occluding loop of bovine cathepsin B and Y59 in the propeptide of HvPap-19, which are potentially involved in a steric clash.

To explain the lack of inhibition of HvPap-19 propeptide on bovine cathepsin B activity, we modelled the structure of both proteins using the crystallographic structure of the human procathepsin B as a template. Bovine cathepsin B and barley cathepsin B HvPap-19 aligned to human procathepsin B at sequence identities of 84.8% and 43.1%, and with Q-MEAN Z-scores of -1.55 and -3.81, respectively. Q-MEAN Z-score is a useful measure for the description of the absolute quality of theoretical models and is a valuable measure for identifying significant errors. Q-MEAN Z-scores less than -4.0 indicate that any part of the protein structure is not modelled correctly. These results imply a very accurate model for bovine cathepsin B and a relatively accurate model for barley HvPap-19. Major differences may be assumed from the models (Fig. 2a). The occluding loop of B cathepsins is clearly higher in the bovine cathepsin B than in HvPap-19. To know if this difference could be a common feature to other animal and plant B cathepsins, an alignment of their complete amino acid sequences was done (Fig. S1). From this alignment, two main conclusions can be reached. First, the occluding loop of plant B cathepsins is shorter than that from animals due to the absence of several amino acid residues in this region (Fig. 2b). Second, an insertion of two amino acid residues in the propeptide region near the occluding loop is conserved in all plant cathepsin B-like sequences (Fig. 2b). The lack of inhibition of a plant cathepsin B propeptide towards an animal cathepsin B can be explained from these two features. In the plant protein, there would not be steric problems between the two additional amino acid residues of the propeptide and the short occluding loop. However, a steric clash would occur between the amino acid residues Y59 of the HvPap-19 propeptide and V174 located at the occluding loop of the bovine cathepsin B (Fig. 2c).

### Structural explanation for differential barley cathepsin L inhibition

**Figure 3:**
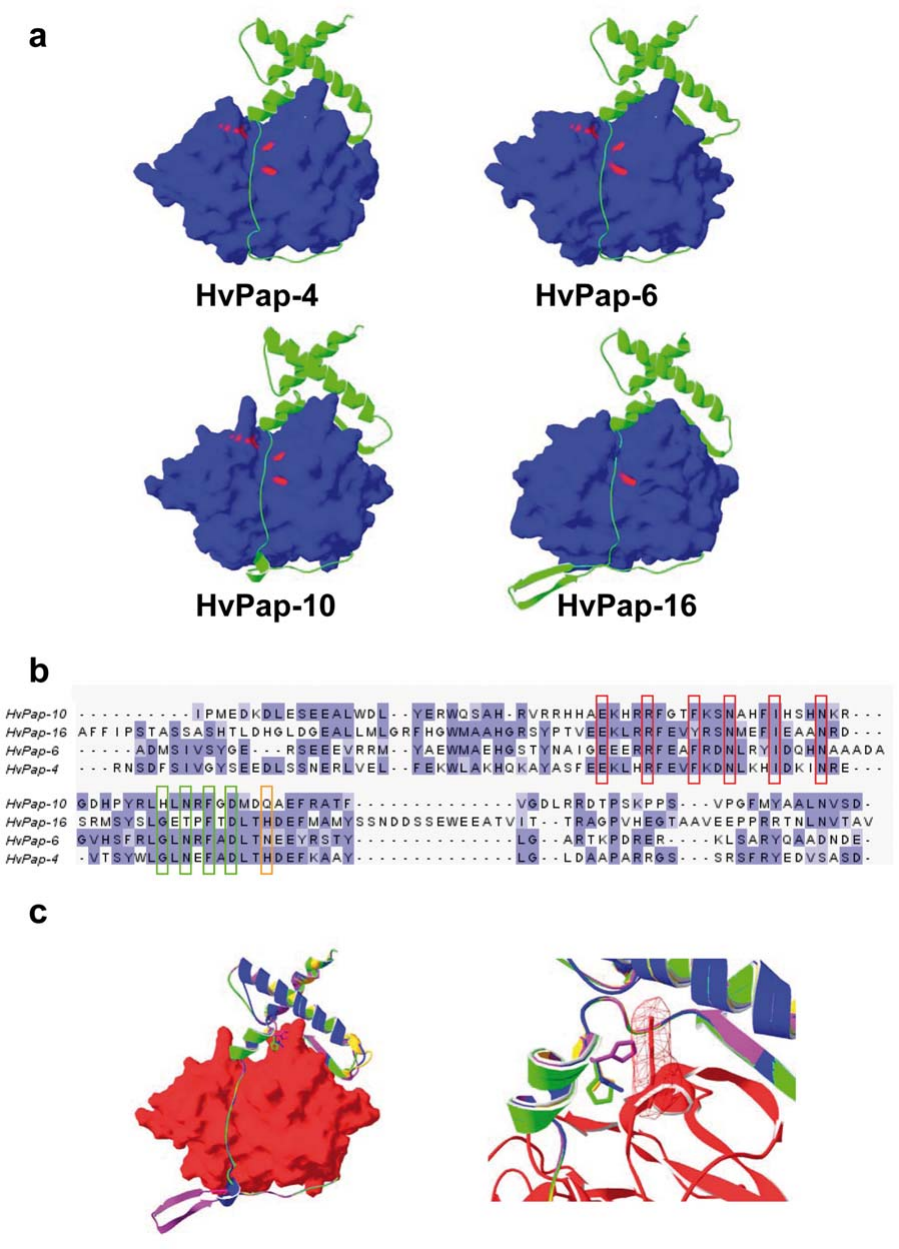
Structure-sequence analysis of barley L-like cathepsins. (a) Homology models of barley cathepsin L-like peptidases created using SWISS-MODEL. The propeptides (green) and their corresponding mature enzymes (blue; catalytic triad residues in red) are shown. (b) Alignment of the amino acid regions of the propeptides from barley L cathepsins. Alignment was generated using the MUSCLE program. The location of residues belonging to the conserved ERFNIN and GNFD motifs (red and green boxes), and the position of a variable key amino acid putatively involved in the interaction propeptide-HvPap-10 enzyme (orange box) are indicated. (c) Ribbon plots showing the structural overlay of three-dimensional models for propeptides from HvPap-4 (green), HvPap-6 (orange), HvPap-10 (blue) and HvPap-16 (purple) peptidases and their interaction with the homology model for HvPap-10 (red). Amino acid residues in position 99 (HvPap-10 numbering) are depicted in stick mode. Close-up image shows the molecular surface of K277 of HvPap-10 peptidase, which is potentially involved in a steric clash with the side chain of H108 of the HvPap-16pro.

To explain the differences of inhibition of barley propeptides on barley cathepsin L-like activities, the structures of the HvPap-4, -6, -10, and -16 proteins were modelled using the crystallographic structure of the papaya cathepsin L-like procaricain as a template (Fig. 3a). Barley HvPap-4, -6, -10, and -16 aligned to procaricain at sequence identities of 50.5%, 46.8%, 43.8% and 34.8%, and with Q-MEAN Z-scores of -3.37, -2.88, -2.91 and -5.47, respectively. These results imply relatively accurate models for barley HvPap-4, -6, and -10 peptidases, and suggest that there is something incorrect in the predicted structure of HvPap-16. A model of HvPap-16 using the mature sequence of the peptidase on the above template had a Q-MEAN Z-score of -3.03, indicating that the HvPap-16 propeptide contributes significantly to the overall model error. An alignment of the propeptide amino acid sequences of the four barley cathepsin L-like proteins show that the conserved propeptide signatures of L cathepsins, the ERFNIN and GNFD motifs, are shared by all (Fig. 3b). However, the propeptide of HvPap-16 has an extension in their C-terminal part in relation to the other propeptides. When the propeptide of HvPap-16 was modelled without the extra amino acid residues that appear in its C-terminal region and forms an additional ß-sheet, the Q-MEAN Z-score was -2.09, which indicates that the wrong part of the molecule should be located in this additional ß-sheet.

Predicted models show some structural differences among the mature enzymes. However, these differences seem not be critical to avoid the interaction among the mature enzymes and the propeptides. Thus, inhibitory *K*_i_ values should be explained by differences in the interaction of some key amino acid residues. An example of how spatial changes in the orientation of the side chain of one amino acid can hinder the interaction propeptide-peptidase in presented (Fig. 3c). HvPap-10 activity is strongly inhibited by HvPap-4pro and HvPap-6pro, weaker by its own HvPap-10 propeptide, and no inhibited by HvPap-16pro. The spatial location of the side chain of amino acids in position 99 (HvPap-10 numbering) can putatively explain these results. HvPap-10 peptidase has a lysine residue at position 277 that located its side chain in the vicinity of the propeptide. At the same position, the four propeptides has different amino acids: Q for HvPap-10, N for HvPap-6, and H for HvPap-4 and -16. Most important is their spatial orientation. As shown in Fig. 2C, the histidine of HvPap-16pro clashes with the molecular surface of K277, the glutamic acid of HvPap-10pro lies near K277, which could difficult the propeptide-enzyme interaction, whereas the side chains asparagine of HvPap-6pro and the histidine of HvPap-4pro are far from the side chain of K277, allowing their full interaction.

**Figure 4:**
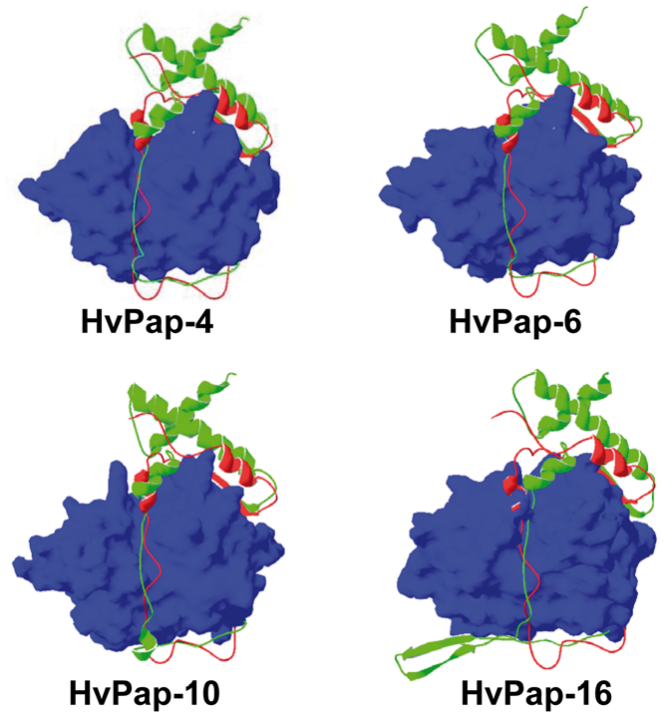
Homology models showing the interaction of the cathepsin B HvPap-19 propeptide with barley L-like cathepsins. Ribbon plots show the structural overlay of the propeptide sequences from HvPap-19 (red) and the barley L-cathepsins (green). Mature peptidases of barley L-cathepsins (HvPap-4, -6, -10 and -16) are coloured in blue.

On the other hand, the propeptide of the cathepsin B-like HvPap-19 was able to inhibit some barley L-cathepsins. This is an unexpected result. In an attempt to explain it, the amino acid structure of HvPap-19pro was superimposed on the model structures of barley L cathepsins using the magic fit tool of the Swiss-PdbViewer program (Fig. 4). However, as magic fit is a tool that permits only an approximation to real structures, major errors could be present. Models indicate that the propeptide of HvPap-19 accommodates with quite accuracy on the structure of the barley cathepsins L, but differences in their inhibitory capacity cannot be deduced from the superimposed structures.

## Discussion

Cysteine peptidases propeptides have been suggested to be potent inhibitors with the ability to control animal parasites and plant pests. Thus, C1A cysteine peptidases of *Trypanosoma cruzi* or *Plasmodium falciparum* (animal parasites) were inhibited by their cognate propeptides [17], [18]. Moreover, the cysteine peptidase activities of midgut crude soluble extracts from Colorado potato beetle or several bruchids (plant pests) were also inhibited by papaya proteinase IV propeptide or by the propeptide of a cysteine peptidase from the bean bruchid *Acanthoscelides obtectus* [19], [20]. Recently, it was demonstrated that genetically modified soybean expressing the propeptide of a C1A peptidase from *Heterodera glycines* (plant nematode pest) reduced soybean cyst nematode infection [21]. Thus, an understanding of the interaction mechanisms involving propeptides and peptidases can allow the development of complementary inhibitors that can achieve broad-spectrum inhibition of parasites and pests.

Propeptides of C1A cysteine peptidases have been shown as tight-binding inhibitors of their cognate enzymes, but also of related peptidases [10]. Thus, to fully address the selectivity of propeptide inhibition both intraspecific and interspecific inhibitory effects has to be considered [4]. To know the capacity of inhibition of plant propeptides, we investigated the selectivity of barley propeptides from C1A cathepsin L and B-like cysteine peptidases. As expected, competitive inhibition was determined to all propeptide-peptidase assays, confirming the putative binding of barley propeptides to the active site of the cysteine peptidases.

As recombinant cathepsins B from plants have not been purified yet, the interspecific inhibitory effects of propeptides on commercial bovine cathepsin B was tested. The occluding loop of cathepsin B has been shown to prevent the propeptide of L-like cathepsins from binding the active site [22], but its intrinsic flexibility permits the interaction with its own propeptide [7]. As expected, barley propeptides from cathepsin L-like peptidases were not able to inhibit bovine cathepsin B. Surprisingly; neither the barley cathepsin B propeptide can inhibit it. Structural features may explain this result. As previously shown [23], the occluding loop of cathepsins B from animals is longer to that of plants. Besides, an insertion in the propeptide sequence of cathepsins B from plants is not present in that from animals. Both features, supported by molecular modelled three-dimensional structures, suggest the existence of steric impediments to enter the plant propeptide into the active site of the bovine cathepsin B.

For cathepsin L-like peptidases, we used the barley members previously purified and characterized [15]. Inhibitory assays indicate that all propeptides are not able to inhibit all barley L cathepsins. Likewise, as occurred in human cathepsin L-like enzymes [24] not all propeptides are better inhibitors of their cognate enzymes than the non cognate ones. Structural features must be involved in the specificity in the interactions. As an example, steric clashes observed in the modelled three-dimensional structures can explain the higher inhibition of HvPap-10 activity exerted by propeptides from HvPap-4 and -6 peptidases than that by its cognate propeptide. In addition, several propeptides can efficiently inhibit peptidases that belong to different cathepsin subfamilies, as the falcipain-2 (cathepsin L-like enzyme) propeptide that is able to inhibit cathepsin L- and B-like peptidases [18]. Similarly, the propeptide of barley cathepsin B-like HvPap-19 was able to inhibit barley cathepsin L-like peptidases.

In conclusion, selectivity of interaction between plant C1A cysteine peptidases and propeptides becomes a complex feature. Molecular modelling of three-dimensional protein structures has become a powerful tool to explain in broad sense the specificity in the interactions. However, as side chain packing is the most difficult part of comparative modelling, further assays should be done to fully understand propeptide/peptidase interactions in order to use plant propeptides as regulators of C1A cysteine peptidases in biotechnological systems.

## Materials and Methods

### Propeptide purification

The cDNA fragments spanning the putative propeptide sequences (HvPap-4pro, R25-D134; HvPap-6pro, A25-E131; HvPap-10pro, I29-D133; HvPap-17pro, A23-V156; HvPap-19pro, A20-Q95) were derived from HvPap-4, -6, -10, and -17 barley genes [3]. These sequences were amplified by PCR and inserted in-frame into the expression vector pRSETB (Invitrogen). Propeptides were expressed and purified as recombinant proteins following the method described in [25]. pRSETB expression plasmids containing the propeptide sequence were introduced into *E. coli* BL21 CodonPlus (Stratagene). Bacterial cells were grown at 37°C to an OD_550_ of ca. 0.5 and induced with 1 mM IPTG (isopropyl β-D-thiogalactopyranoside) for 2 h 30 min, harvested and processed. The fusion proteins with the histidine tail were purified using a His-Bind Resin (Novagen) following the manufacturer's instructions. Purification process was checked by SDS-PAGE. The final protein concentration was quantified by the BioRad kit with bovine serum albumin (BSA) as standard. Additional MALDI-TOF analysis was performed to check molecular mass and propeptide identity by peptide mass fingerprint after trypsin digestion.

### Inhibitory assays

Recombinant barley HvPap-4, -6, -10, -16 cysteine peptidases were purified and activated from *E. coli* cultures as described [15]. The recombinant propeptides were assayed against these peptidases and commercial bovine cathepsin B (Calbiochem). Briefly, different concentrations of propeptides plus the corresponding peptidase were incubated in a buffer containing 100 mM sodium phosphate pH 6.0, 10 mM L-cysteine, 10 mM EDTA and 0.01% (v/v) Brij35 at room temperature for 10 min. Then, the Z-FR-AMC (for cathepsin L) or Z-RR-AMC (for cathepsin B) fluorescent substrates were added and the reactions were incubated for 1 h at 30°C. Emitted fluorescence was measured with a microplate fluorescence reader (Tecan GeniusPro) using an excitation filter of 365 nm and an emission filter of 465 nm. The system was calibrated with known amounts of AMC hydrolysis product in a standard reaction mixture. All assays were carried out in triplicate and blanks were used to account for the spontaneous breakdown of substrates. As negative control, proteins from *E. coli* transformed with the empty expression vector were used. Enzyme concentrations were determined by active-site titration with the irreversible inhibitor L-trans-Epoxysuccinyl-leucylamido(4-guanidino)butane (E-64). Similarly, the concentration of correctly folded propeptides was determined by titration with different barley papain-like peptidases previously titrated with E-64. The kind of inhibition was determined from Lineweaver-Burk plots (1/V versus 1/[S]), and confirmed by lineal correspondence between IC_50_ and [E] values and IC_50_ and [S] values [26]. Apparent *K*_i_ [*K*_i(app)_] values were calculated by non-linear regression using the GraFit program [27], according to the Morrison equation. The inhibition constants (*K*_i_) were then calculated according to the equation *K*_i_ = *K*_i(app)_/(1+[S]/*K*_m_) using *K*_m_ values calculated by non-linear regression of the Michaelis-Menten equation using the GraFit program.

### Alignments of propeptide/peptidase sequences

The amino acid sequences of barley C1A cysteine peptidases were extracted from the NCBI GenBank. BlastP searches for cathepsin B-like cysteine peptidases were made using the amino acid sequence of the HvPap-19 protein [3]. Animal and plant proteins that conserve the specific features for cathepsin B peptidases were selected. Information about protein models is compiled in Table S1. Alignments of the amino acid sequences were performed using the default parameters of MUSCLE [28]. Depicted alignments were obtained by the multiple alignment editor Jalview version 2.6 [29].

### Molecular modelling of propeptide-cathepsin interaction

The three-dimensional structures of the barley cysteine peptidases and the bovine cathepsin B were modelled using the standard automated routine of SWISS-MODEL program [30]. The known crystal structures of the cathepsin L-like peptidase from papaya, procaricain (PDB identifier 1PCI) and the cathepsin B from human (PDB identifier 3PBH) were used to construct the homology-based models. The template structures were selected on the basis of highest sequence similarities. Models were evaluated with the QMEAN Z-score for predicting the absolute quality of a model [31]. The Swiss-PdbViewer program [32] was used to generate the single and superimposed images of protein models.

## Supporting Information

Figure S1 **Comparison of the amino acid sequences of the cathepsin B-like cysteine proteases.** The alignment was generated using the MUSCLE program. Pp, *Physcomitrella patens*; Sm, *Selaginella moellendorffii*; Os, *Oryza sativa*; Hv, *Hordeum vulgare*; Pt, *Populus trichocarpa*; At, *Arabidopsis thaliana*.
(DOC)

Table S1 **Information about the cathepsin B and L-like proteins used in the alignments.**
(DOC)
