# Supplementary material for: Structural Basis for Specificity of Propeptide-Enzyme Interaction in Barley C1A Cysteine Peptidases
Source: PLoS One. 2012 May 17;7(5):e37234. doi: 10.1371/journal.pone.0037234 (PMC3355106; doi:10.1371/journal.pone.0037234)
Supplement: Figure S1 — Comparison of the amino acid sequences of the cathepsin B-like cysteine proteases. The alignment was generated using the MUSCLE program. Pp, Physcomitrella patens; Sm, Selaginella moellendorffii; Os, Oryza sativa; Hv, Hordeum vulgare; Pt, Populus trichocarpa; At, Arabidopsis thaliana. (DOC) [file pone.0037234.s001.doc]

**Supporting Figure.**

**Figure S1.** Comparison of the amino acid sequences of the cathepsin B-like cysteine proteases. The alignment was generated using the MUSCLE program. Pp, *Physcomitrella patens*; Sm, *Selaginella moellendorffii*; Os, *Oryza sativa*; Hv, *Hordeum vulgare*; Pt, *Populus trichocarpa*; At, *Arabidopsis thaliana*.

CathB_Zebrafish --------MWR-LAFLCVISALS----------------VSWARPRLAPLSHEMVNFINK

CathB_Frog --------MWHLVVALCFLASIA----------------NSRHLPYFAPLSHDMVNYINK

CathB_Chicken --------MSWSRSILCLLGAFA----------------NARSIPYYPPLSSDLVNHINK

CathB_Mice --------MWWSLILLSCLLALT----------------SAHDKPSFHPLSDDLINYINK

CathB_Human --------MWQLWASLCCLLVLA----------------NARSRPSFHPLSDELVNYVNK

CathB_Dog --------MWQLLTTLSCLVMLT----------------GAQSRLPFRALSDELVDYVNK

CathB_Horse --------MWQLLATLSCLVVLT----------------NARSRPYFQPLSDELVNYVNK

CathB_Pig --------MWRLLATLSCLVLLT----------------SARESLHFQPLSDELVNFINK

CathB_Cow --------MWRLLATLSCLLVLT----------------SARSSLYFPPLSDELVNFVNK

PpPap-9 --------MKPISLLLLCSVILA------AQAARVEPDLLESKRLIHQQL---LVDKVNA

SmPap-19 --------MESSLVLAAAAIALL----FSAVAQGVRVAESGKLDLGRPLLQKSIVDIVNN

OsPap-45 --------MGMPLLLLLLILVAAGPQAGRAAKPIPNLQLMTKEGGSSRIIQDDIIKAINK

HvPap-19 --------MGSGLLPLALLVVLS------AAAAAPQLAGAAGGGHSLGIIQKGIIQTVNN

HvPap-20 -----MGGVGVSLALLAAAAAVV----ISAVAAAPQLVGAAKAEHSLGIIQEDIIQTVND

PtPap-1 ---METSLCFSTLLLLLIGAIFT------FQSQVIAVEPVSDLKLNSRILQDSILKKVNG

AtPap-29 MADNCIRLLHSASVFFCLGLLIS----SFNLLQGIAAENLSKQKLTSWILQNEIVKEVNE

AtPap-30 -MAVYNTKLCLASVFLLLGLLLA------FDLKGIEAESLTKQKLDSKILQDEIVKKVNE

: ::. :*

CathB_Zebrafish A-NTTWTAGHN--FRDVDYSYVKRLCGTFLKGPK-----LPVMVQYTEGLKLPKNFDARE

CathB_Frog V-NTTWKAGHN--FANADVHYVKRLCGTHLNGPQ-----LQKRFGFADDLDLPDSFDSRA

CathB_Chicken L-NTTGRAGHN--FHNTDMSYVKKLCGTFLGGPK-----APERVDFAEDMDLPDTFDTRK

CathB_Mice Q-NTTWQAGRN--FYNVDISYLKKLCGTVLGGPK-----LPGRVAFGEDIDLPETFDARE

CathB_Human R-NTTWQAGHN--FYNVDMSYLKRLCGTFLGGPK-----PPQRVMFTEDLKLPASFDARE

CathB_Dog R-NTTWKAGHN--FHNVDPSYLRRLCGTFLGGPK-----LPQRVQFAKNLILPESFDARE

CathB_Horse R-NTTWKAGHN--FHNVDLSYVKRLCGTFLGGPK-----LPQRVWFAEDVVLPENFDARE

CathB_Pig Q-NTTWTAGHN--FYNVDLSYVKKLCGTFLGGPK-----LPQRAAFAADMILPKSFDARE

CathB_Cow Q-NTTWKAGHN--FYNVDLSYVKKLCGAILGGPK-----LPQRDAFAADVVLPESFDARE

PpPap-9 HPRATWKAGFNDRFEGHTIEHLKKICGAKMTPANELEPSIERVTHKHKKLVLPKEFDARK

SmPap-19 DPNAGWKAGFNERFINHTVRDFKRLCGVLPKSSEEVQPLRPLRSH-PRTLDLPKHFDARE

OsPap-45 HPNAGWTAARNPYFANYTTAQFKHILGVKPTPHSVLN-DVPVKTY-PRSLMLPKEFDARS

HvPap-19 HPNAGWTAGHNPYLANYTIEQFKHMLGVKPTPPGLLA-GVRTKTH-PRSEQLPKEFDARS

HvPap-20 HPNAGWTAGHNPYFANYTIEQFKHILGVKPTPPGLLA-GVPIKTH-PKSADLPKEFDART

PtPap-1 NPKAGWKATMNHHFSNYTVAQFKYLLGVKPTPKEELR-GIPVISH-PKSLRLPEEFDART

AtPap-29 NPNAGWKASFNDRFANATVAEFKRLLGVKPTPKTEFL-GVPIVSH-DISLKLPKEFDART

AtPap-30 NPNAGWKAAINDRFSNATVAEFKRLLGVKPTPKKHFL-GVPIVSH-DPSLKLPKAFDART

.: * * : . .. : *. ** **:*

CathB_Zebrafish QWPNCPTLKEIRDQGSCGSCWAFGAAEAISDRVCIQSNAKVSVEISSQDLLTCCD-SCGM

CathB_Frog AWPNCPTIREIRDQGSCGSCWAFGAVEAISDRVCVHTNGKVNVEVSAEDLLSCCGFKCGM

CathB_Chicken QWPNCPTISEIRDQGSCGSCWAFGAVEAISDRICVHTNAKVSVEVSAEDLLSCCGFECGM

CathB_Mice QWSNCPTIGQIRDQGSCGSCWAFGAVEAISDRTCIHTNGRVNVEVSAEDLLTCCGIQCGD

CathB_Human QWPQCPTIKEIRDQGSCGSCWAFGAVEAISDRICIHTNAHVSVEVSAEDLLTCCGSMCGD

CathB_Dog QWPNCPTIKEIRDQGSCGSCWAFGAVEAISDRICIRTNGHVNVEVSAEDMLTCCGDQCGD

CathB_Horse QWPNCPTIKEIRDQGSCGSCWAFGAVEAISDRICIRTNGHVSVEVSAEDMLTCCGDQCGD

CathB_Pig QWPNCPTIKEIRDQGSCGSCWAFGAVEAISDRICIRSNGRVNVEVSAEDMLTCCGDECGD

CathB_Cow QWPNCPTIKEIRDQGSCGSCWAFGAVEAISDRICIHSNGRVNVEVSAEDMLTCCDGECGD

PpPap-9 HWGHCSTIGAILDQGHCGSCWAFGAAESLTDRFCIHMN--ESVSLSENDLLACCGFECGD

SmPap-19 AWPQCSSIKNILDQGHCGSCWAFGAVEALTDRFCILNN--ENVSLSENDLVACCS-SCGF

OsPap-45 AWSQCNTIGTILDQGHCGSCWAFGAVECLQDRFCIHFN--MNISLSVNDLVACCGFMCGD

HvPap-19 KWSGCSTIGKILDQGHCGSCWAFGAVECLQDRFCIHHN--MNISLSANDLVACCGFMCGD

HvPap-20 QWSSCSTIGNILDQGHCGACWAFAAVESLQDRFCIHLN--MSVSLSVNDLLACCGFLCGS

PtPap-1 AWPQCSTIGKILDQGHCGSCWAFGAVESLSDRFCIHYG--MNISLSVNDLLACCGFLCGS

AtPap-29 AWSQCTSIGRILDQGHCGSCWAFGAVESLSDRFCIKYN--MNVSLSVNDLLACCGFLCGQ

AtPap-30 AWPQCTSIGNILDQGHCGSCWAFGAVESLSDRFCIQFG--MNISLSVNDLLACCGFRCGD

* * :: * *** **:****.*.*.: ** *: . .:.:* :*:::**. **

CathB_Zebrafish GCNGGYPSAAWDFWTTDGLVTGGLYNSHIGCRPY-TIEPCEHHVNGSRPPCTGEGGDTPN

CathB_Frog GCNGGYPSGAWRFWTETGLVSGGLYDSHVGCRPY-SIPPCEHHVNGSRPSCKGEEGDTPK

CathB_Chicken GCNGGYPSGAWRYWTERGLVSGGLYDSHVGCRAY-TIPPCEHHVNGSRPPCTGEGGETPR

CathB_Mice GCNGGYPSGAWSFWTKKGLVSGGVYNSHVGCLPY-TIPPCEHHVNGSRPPCTGE-GDTPR

CathB_Human GCNGGYPAEAWNFWTRKGLVSGGLYESHVGCRPY-SIPPCEHHVNGSRPPCTGE-GDTPK

CathB_Dog GCNGGFPAEAWNFWTKQGLVSGGLYDSHVGCRPY-SIPPCEHHVNGSRPPCTGE-GDTPK

CathB_Horse GCNGGFPAEAWNFWTKQGLVSGGLYDSHVGCRPY-SIPPCEHHVNGSRPPCTGEGGDTPK

CathB_Pig GCNGGFPSGAWNFWTKKGLVSGGLYDSHVGCRPY-SIPPCEHHVNGSRPPCTGE-GDTPK

CathB_Cow GCNGGFPSGAWNFWTKKGLVSGGLYNSHVGCRPY-SIPPCEHHVNGSRPPCTGE-GDTPK

PpPap-9 GCDGGYPIRAWRYFKRTGVVTS-------KCDPYFDQIGCGH------PGCYPT-YRTPK

SmPap-19 GCDGGYPYAAWEYFAQTGVVTS-------QCDPYFDGKGCKH------PGCEPE-YDTPV

OsPap-45 GCDGGYPIMAWRYFVRNGVVTD-------ECDPYFDQVGCKH------PGCEPA-YPTPV

HvPap-19 GCDGGYPISAWQYFVQNGVVTE-------ECDPYFDQVGCKH------PGCEPA-YPTPV

HvPap-20 GCNGGYPISAWRYFRRSGVVTE-------ECDPYFDQTGCQH------PGCEPA-YPTPK

PtPap-1 GCNGGYPISAWRYFVHHGVVTE-------ECDPYFDDIGCSH------PGCEPG-YPTPK

AtPap-29 GCNGGYPIAAWRYFKHHGVVTE-------ECDPYFDNTGCSH------PGCEPA-YPTPK

AtPap-30 GCDGGYPIAAWQYFSYSGVVTE-------ECDPYFDNTGCSH------PGCEPA-YPTPK

**:**:* ** :: *:*: * .* * * * * **

CathB_Zebrafish CDMKCEPGYSPLYKEDKHFGKTSYSVPSNQNGIMAELFKNGPVEAAFTVYEDFLLYKSGV

CathB_Frog CMKTCEEGYTPAYGSDKHFGATSYGVPSSEKEIMADIYKNGPVEGAFVVYADFPLYKSGV

CathB_Chicken CSRHCEPGYSPSYKEDKHYGITSYGVPRSEKEIMAEIYKNGPVEGAFIVYEDFLMYKSGV

CathB_Mice CNKSCEAGYSPSYKEDKHFGYTSYSVSNSVKEIMAEIYKNGPVEGAFTVFSDFLTYKSGV

CathB_Human CSKICEPGYSPTYKQDKHYGYNSYSVSNSEKDIMAEIYKNGPVEGAFSVYSDFLLYKSGV

CathB_Dog CSKICEPGYSPSYKEDKHYGCSSYSVSDNEKEIMAEIYKNGPVEAAFTVYSDFLLYKSGV

CathB_Horse CSKICEPGYSPSYKEDKHYGCSSYSVSSSEKEIMAEIFKNGPVEAAFTVYSDFLQYKSGV

CathB_Pig CSKICEPGYTPSYKEDKHFGCSSYSISRNEKEIMAEIYKNGPVEGAFTVYSDFLQYKSGV

CathB_Cow CSKTCEPGYSPSYKEDKHFGCSSYSVANNEKEIMAEIYKNGPVEGAFSVYSDFLLYKSGV

PpPap-9 CVKHCVD--DELWVKSKHLSVNAYEVSKEPEDLMAELYTNGPIEVSFEVFEDFAHYKTGV

SmPap-19 CVKQCVD--NEQWRDSKHFTVQTYAVNSDIYDIQAEIYKNGPVEVSYTVYEDFAHYKSGV

OsPap-45 CEKKCKVQ-NQVWLEKKHFSVNAYRVNSDPHDIMAEVYQNGPVEVAFTVYEDFAHYKSGV

HvPap-19 CEKKCKVQ-NQVWQEKKHFSIDAYQVNSDPHDIMAEVYKNGPVEVAFTVYEDFAHYKSGV

HvPap-20 CHRKCKVE-NQVWKKNKHFSVNAYRVHSNPHDIMAEVYKNGPVEVAFTVYEDFAHYKSGV

PtPap-1 CARKCVNK-NQLWKKSKHYGVKPYRIDSDPESIMAEIYKNGPVEVAFTVYEDFAHYKSGV

AtPap-29 CARKCVSG-NQLWRESKHYGVSAYKVRSHPDDIMAEVYKNGPVEVAFTVYEDFAHYKSGV

AtPap-30 CSRKCVSD-NKLWSESKHYSVSTYTVKSNPQDIMAEVYKNGPVEVSFTVYEDFAHYKSGV

* * : ..** .* : : *::: ***:* :: *: ** **:**

CathB_Zebrafish YQHMSGSALGGHAIKILGWGE-ENGVPYWLAANSWNTDWGDNGYFKILRGEDHCGIESEI

CathB_Frog YQHETGEELGGHAIKILGWGV-ENGTPYWLCANSWNTDWGDNGFFKILRGKDHCGIESEV

CathB_Chicken YQHVSGEQVGGHAIRILGWGV-ENGTPYWLAANSWNTDWGITGFFKILRGEDHCGIESEI

CathB_Mice YKHEAGDMMGGHAIRILGWGV-ENGVPYWLAANSWNLDWGDNGFFKILRGENHCGIESEI

CathB_Human YQHVTGEMMGGHAIRILGWGV-ENGTPYWLVANSWNTDWGDNGFFKILRGQDHCGIESEV

CathB_Dog YQHVTGEMMGGHAVRILGWGV-EDGTPYWLVGNSWNTDWGDNGFFKILRGRDHCGIESEI

CathB_Horse YQHVAGDMMGGHAVRILGWGV-ENGTPYWLVGNSWNTDWGDNGFFKILRGQDHCGIESEI

CathB_Pig YQHVTGDLMGGHAIRILGWGV-ENGTPYWLVGNSWNTDWGDNGFFKILRGQDHCGIESEI

CathB_Cow YQHVSGEIMGGHAIRILGWGV-ENGTPYWLVGNSWNTDWGDNGFFKILRGQDHCGIESEI

PpPap-9 YKHVYGRYIGGHAVKLIGWGTTDDGVDYWTIVNSWNTNWGEHGLFRIARGGNECGIESYA

SmPap-19 YKHVFGEVLGGHAVKFIGWGTTDDGKDYWIVANSWNRSWGEDGFFQISRGSNECGIESEP

OsPap-45 YKHITGGMMGGHAVKLIGWGTTDAGEDYWLLANQWNRGWGDDGYFKIIRGTNECGIEEDV

HvPap-19 YKHITGGVMGGHAVKLIGWGTSDAGEDYWLLANQWNRGWGDDGYFKIIRGKNECGIEEDV

HvPap-20 YKHITGGVMGGHAVKLIGWGTSDAGEDYWLLANQWNRGWGDDGYFKIIRGKNECGIEEDV

PtPap-1 YKHITGGMMGGHAVKLIGWGTSEDGEAYWLLANQWNRGWGDDGYFKIRRGTNECGIEGDV

AtPap-29 YKHITGTNIGGHAVKLIGWGTSDDGEDYWLLANQWNRSWGDDGYFKIRRGTNECGIEHGV

AtPap-30 YKHITGSNIGGHAVKLIGWGTSSEGEDYWLMANQWNRGWGDDGYFMIRRGTNECGIEDEP

*:* * :****:.::*** . * ** *.** .** * * * ** : ****

CathB_Zebrafish VAGIPM-------------------

CathB_Frog VAGIPKN------------------

CathB_Chicken VAGVPRMEQYWTRV-----------

CathB_Mice VAGIPRTDQYWGRF-----------

CathB_Human VAGIPRTDQYWEKI-----------

CathB_Dog VAGIPCTDQYWKKI-----------

CathB_Horse VAGIPCTDQYWKRI-----------

CathB_Pig VAGIPCTPHF---------------

CathB_Cow VAGMPCTHQY---------------

PpPap-9 VAGLPFDKGLHSAM-----------

SmPap-19 VAGIPLKKTGFSDI-----------

OsPap-45 VAGMPSTKNMVRNYDSAFGVGTAIV

HvPap-19 TAGMPSMKNIAGSAFAM--------

HvPap-20 TAGMPSTKNMDRNNDVAF--GTAIL

PtPap-1 VAGLPSTRNLVREVVSVDAREDASA

AtPap-29 VAGLPSDRNVVKGITTSDDLLVSSF

AtPap-30 VAGLPSSKNVFRVDTGSNDLPVASV

.**:*
