# Supplementary material for: Structural Basis for Specificity of Propeptide-Enzyme Interaction in Barley C1A Cysteine Peptidases
Source: PLoS One. 2012 May 17;7(5):e37234. doi: 10.1371/journal.pone.0037234 (PMC3355106; doi:10.1371/journal.pone.0037234)
Supplement: Table S1 — Information about the cathepsin B and L-like proteins used in the alignments. (DOC) [file pone.0037234.s002.doc]

**Supporting Table.**

**Table S1.** Information about the cathepsin B and L-like proteins used in the alignments.

| **Protein** | **Organism** | **Gene model/Accesion number** |
| --- | --- | --- |
| CathB_Human | *Homo sapiens* | NP_001899 |
| CathB_Dog | *Canis familiaris* | XP_543203 |
| CathB_Horse | *Equus caballus* | XP_001498242 |
| CathB_Pig | *Sus scrofa* | NP_001090927 |
| CathB_Cow | *Bos taurus* | AAI02998 |
| CathB_Mice | *Mus musculus* | NP_031824 |
| CathB_Chicken | *Gallus gallus* | NP_990702 |
| CathB_Zebrafish | *Danio rerio* | AAH44517 |
| CathB_Frog | *Xenopus laevis* | NP_001080410 |
| PpPap-9 | *Physcomitrella patens* | estExt_Genewise1.C_790115 |
| SmPap-19 | *Selaginella moellendorffii* | estExt_fgenesh1_pm.C_1500002 |
| OsPap-45 | *Oryza sativa* | Os05g24550 |
| HvPap-19 | *Hordeum vulgare* | AJ310426 |
| HvPap-20 | *Hordeum vulgare* | AM941127 |
| PtPap-1 | *Populus trichocarpa* | eugene3.00021714 |
| AtPap-29 | *Arabidopsis thaliana* | At1g02305 |
| AtPap-30 | *Arabidopsis thaliana* | At4g01610 |
| HvPap-4 | *Hordeum vulgare* | AM941118 |
| HvPap-6 | *Hordeum vulgare* | AM941120 |
| HvPap-10 | *Hordeum vulgare* | U19384 |
| HvPap-16 | *Hordeum vulgare* | AM941126 |
